# Malaria and Its Cause and Effect

**Published:** 1855-12

**Authors:** C. D. Griswold


*Malaria and its Cause and Effect.* By C. D. Griswold, M. D.

ARTICLE FIRST.

If the amount written upon the subject of malaria, and its cause and effects,could be considered an argument against its further discussion, I should not employ my pen in adding to the already numerous treatises which occupy a large space in medical literature. Much as has been written upon this subject, I believe it is generally conceded that but few authors have succeeded in giving the necessary practical information for the treatment of the disease; and in a still greater degree have they mostly failed to point out clearly the cause, and the necessary means for its prevention.

For this reason probably more than any other, the subject has long since failed to attract that interest which its importance demands.

That form of malarial fever known as intermittent has been regarded the type of all fevers; presenting the most perfectly marked phenomenon in its different stages, which are common, in a more or less perfect degree, in every variety. The study, therefore, of this pure form of fever is absolutely essential to a thorough knowledge of this protean class of maladies.

Believing, as I do, that all varieties of the non-contagious fevers arise from different conditions of the same cause, the intermittent, being the simplest and purest of any, is to me invested with greater interest and importance than it can be to those who think differently.

Most of the elaborate treatises upon the acknowledged varieties of malarial fever are made up from the observations of men of limited experience—men who only had the opportunity of investigating the disease in a particular climate or situation; and hence it is that conflicting opinions have prevailed to such a degree that we have no well-established doctrines for our guide. Every disease varies more or less in its characteristics in different latitudes, and as malarial fevers are dependent upon terrestrial emanations, which are subject to different conditions of temperature in the widely different parts of the world where they prevail, it must necessarily follow that they are more likely to assume a greater variety of forms than any other. Such being the fact, I come to a ready explanation of what I deem another fact, namely, that the same order of fever, arising from the same cause, varying only from climactric conditions, is known by different names, often implying different pathological conditions and methods of treatment. Besides the diseases known or recognized as fevers, there is also a variety of maladies known by different names, presenting widely diversified symptoms, which are traceable directly to the same cause for their origin, and dependent upon constitutional idiosyncracies for their anomalous character.

Should any be disposed to maintain—as many, no doubt, there are—that this subject has already been sufficiently discussed, I have but to refer to the fact that a great majority of the diseases the physician is called upon to treat are of a malarial origin; and that they extend over a wider portion of the globe than any other—that many portions of our own country, and the entire belt of the inter-tropics, are rendered almost uninhabitable by the civilized races from the effects of malarial poison; and that in and about New York malarial fevers are more prevalent at the present time than they were ever known before. If, in connection with these facts, we consider the additional fact that we have a remedy which approaches the nearest to a specific of anything known in the whole range of medicaments, we must conclude that our knowledge, or its application, is not equal to the demand.

I would not assert that this subject has never been understood—far from it; on the contrary, there is perhaps not a point in relation to malaria and its cause and effects but what has been accurately treated. The difficulty is simply this, with the great amount of truth a vast deal of error has been incorporated, so that even the condensed treatises of our text books are rendered exceedingly uncertain guides; and therefore the physician without a larger experience than usually falls to the lot of one man, coupled with accurate habits of observation, may go through life without being able to correct the errors inculcated in his preliminary education.

While many portions of the world which are richest in natural resources are rendered unfit for the habitation of the human race from the prevalence of malaria, it certainly cannot be claimed that there is no further object for investigation.

Were it not for this one pestiferous agent, that portion of the globe lying between the tropics would be far preferable for the maintenance of the human race than northern latitudes. Exempt as it is from that great scourge of the north, consumption, vastly superior in natural productions, with a climate so unchanging that little care is essential to provide against it, it would seem that the curse upon Adam had been visited upon the whole human race in thus driving us from this garden of the world by these noxions emanations, to sweat the brow during one portion of the year, in providing for the barrenness of the next. It is as much within the province of our science to provide against disease as for the treatment of it; yet, in relation to measures for protection against the pro. duction of malaria, we certainly have done very little.

New York and its environs, embracing a population of one million, the boasted central point of commerce and of improvement, suffers annually beyond calculation from the effects of malaria. Throughout our most densely populated streets malarial fevers are by no means uncommon, and yet it requires but the application of the simplest principles of medical science to remove the cause entirely.

Another consideration, apart from that of the public health, it may not be improper to urge here, as it is one which never fails to excite interest and lead to action. It is well known how detrimental an aguish reputation is to the pecuniary interest of a community. Motives, therefore, of private interest would be quite sufficient to cause the necessary sanitary measures to be entered into for the prevention of this class of diseases in every community, where the soil is valuable, if the means necessary were generally understood. Upon the subject of the source of malaria the grossest ignorance generally prevails; and I regret to say, that within the range of my experience, physicians are about as much in error as the public. Whether it is that they have not sufficient experience to enable them to discriminate between erroneous theories and the true cause, or that they do not take pains to make the necessary observations, it would not become me to say, but certain it is that the most erroneous notions are often disseminated by them. An example of this is the very prevalent opinion on our sea borders that malaria is developed from the salt water marshes. Now as salt water marshes are not to be remedied in many instances, such a doctrine as this is at once fatal to the hopes of those who desire the improvement of the district bordering on them. One of the most delightful districts adjacent to New York has been cursed by the inculcation of this error for the last forty years. An arm of the sea, situated at a distance of a mile and a half, overflowing at high tide a portion of lowland, has been considered the cause of the annual prevalence of fevers over a portion of country extending far beyond the reach of the most virulent malaria. A proper understanding of the subject, with the expenditure of a few thousand dollars in draining and filling in a few little ponds that have existed since the country was first inhabited, would have rendered it not only one of the most healthy, but delightful places of residence near our city.

This is one of the many examples that could be cited to show, not only how the public health may be impaired, but also how pecuniary interest suffers from an ignorance of the cause of malaria.

The laws which govern health are but little understood by the non-professional public, and therefore measures involving great expense are not readily entered into solely upon such considerations. On the other hand, however, when a man learns that his property will be enhanced in value in a ten-fold proportion on a certain outlay, he eagerly grasps at the idea, and wishes for a full understanding of the subject.

To demonstrate so far as practicable the laws which govern the production of malaria, and show how easily the cause may be removed in many instances, and the advantages to health and pecuniary interest resulting therefrom, is one of the chief objects of this series of articles. In doing so I shall be confined principally to my own observations in this country and the inter-tropics, without much reference to the views of other writers, whether agreeing with them or not.—*American Medical Gazette and Journal of Health.*

